# Skeletal Muscle Mitochondria and Aging: A Review

**DOI:** 10.1155/2012/194821

**Published:** 2012-07-19

**Authors:** Courtney M. Peterson, Darcy L. Johannsen, Eric Ravussin

**Affiliations:** Department of John S. Mclhenny Skeletal Muscle Physiology, Pennington Biomedical Research Center, 6400 Perkins Road, Baton Rouge, LA 70808, USA

## Abstract

Aging is characterized by a progressive loss of muscle mass and muscle strength. Declines in skeletal muscle mitochondria are thought to play a primary role in this process. Mitochondria are the major producers of reactive oxygen species, which damage DNA, proteins, and lipids if not rapidly quenched. Animal and human studies typically show that skeletal muscle mitochondria are altered with aging, including increased mutations in mitochondrial DNA, decreased activity of some mitochondrial enzymes, altered respiration with reduced maximal capacity at least in sedentary individuals, and reduced total mitochondrial content with increased morphological changes. However, there has been much controversy over measurements of mitochondrial energy production, which may largely be explained by differences in approach and by whether physical activity is controlled for. These changes may in turn alter mitochondrial dynamics, such as fusion and fission rates, and mitochondrially induced apoptosis, which may also lead to net muscle fiber loss and age-related sarcopenia. Fortunately, strategies such as exercise and caloric restriction that reduce oxidative damage also improve mitochondrial function. While these strategies may not completely prevent the primary effects of aging, they may help to attenuate the rate of decline.

## 1. Introduction

Around the fourth decade of life, both muscle mass and strength begin to decline [1], and these declines accelerate with advancing age [2]. The loss of muscle mass occurs at a rate of just under 1% per year [3] and appears to be an unavoidable consequence of aging, although it can be slowed by exercise, especially resistance training [4–6]. A significant concern is that as one ages, changes in muscle mass and strength tend to be dissociated. Data from the Baltimore Longitudinal Study of Aging [7] and the Health ABC study [3] showed using DXA and CT that muscle strength declined three times faster than muscle mass, suggesting a decrease in muscle “quality.” This posits that along with an overall reduction in tissue mass, changes are occurring within the skeletal muscle to affect strength. Changes such as accumulation of intra- and extra-myocellular lipids, improper folding of structural and contractile proteins, and mitochondrial dysfunction are thought to occur with age and are the topic of intense scrutiny [8–10].

**Figure 1:**
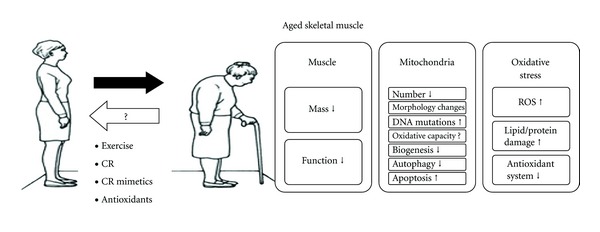
This cartoon describes the changes in skeletal muscle with aging on the right side of the figure. Both the mass and function of skeletal muscle are decreased in elderly people. Furthermore, at the mitochondrial level, the number of mitochondria is decreased in parallel with changes in mitochondrial morphology. Mitochondrial DNA, oxidative capacity, biogenesis, and autophagy are all decreased in conjunction with an increased number of DNA mutations and increased levels of apoptosis. Finally, oxidative stress is increased in the muscles of elderly people in association with cellular lipid, protein, and DNA damage. The bottom left of the cartoon shows that exercise, caloric restriction, caloric restriction mimetics, and antioxidants can all delay the aging of skeletal muscle.

Dysfunctional mitochondria in particular are thought to play a key role in muscle function decline, as the mitochondria are the main producers of both cellular energy and free radicals. Alterations in mitochondria have been noted in aging, including decreased total volume, increased oxidative damage, and reduced oxidative capacity. These biochemical and bioenergetic changes are accompanied by perturbations in cellular dynamics, such as a decrease in mitochondrial biogenesis and an increase in mitochondrially mediated apoptosis (Figure 1). These changes may underlie not only a loss of muscle quality with age, but also other common age-associated pathologies such as ectopic lipid infiltration, systemic inflammation, and insulin resistance [10]. In this paper, we examine the evidence for age-related changes in skeletal muscle mitochondria, with a focus on studies conducted in humans.

### 1.1. Overview of Mitochondria

Mitochondria are double-membrane encoded organelles with their own genome that consume oxygen and substrates to generate the vast majority of ATP while producing reactive oxygen species in the process. They also participate in a wide range of other cellular processes, including signal transduction, cell cycle regulation, oxidative stress, thermogenesis, and apoptosis. In doing so, they are highly dynamic organelles that are continuously remodeling through biogenesis, fission, fusion, and autophagy, thus responding to and modulating cellular dynamics. For instance, they undergo biogenesis to meet increased energy demands in response to exercise, and they ensure cellular quality by initiating an apoptotic program to remove defective cells.

**Figure 2:**
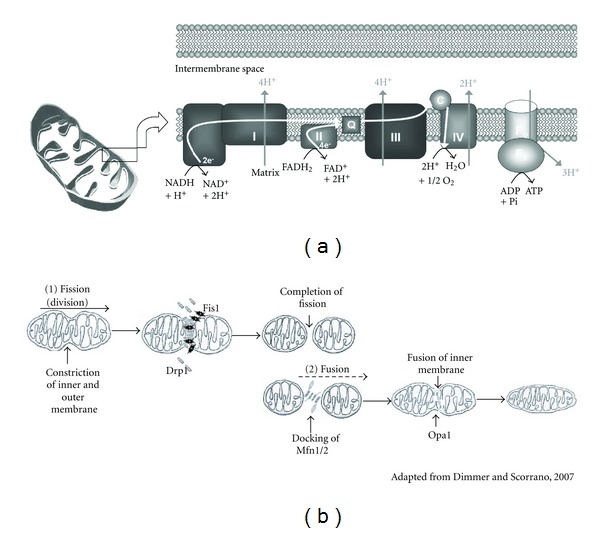
Mitochondrial processes are both static (a) and dynamic (b). (a) depicts the classical movement of electrons along complexes I–IV embedded in the inner mitochondrial membrane with the generation of a proton gradient (membrane potential). The proton gradient causes hydrogen ions to flow back into the mitochondrial matrix via complex V (ATP synthase), producing ATP in the process. (b) depicts the processes of mitochondrial fusion and fission. Mitochondria can undergo constriction and division (1), mediated by Drp1, which bonds and localizes to the constriction site via an interaction with the receptor-like protein Fis1 (2). During fusion, a tether of the Mfn1/2 to collateral mitochondrial Mfn1/2 conjoins the outer membranes. Opa1, an inner membrane GTPase protein, facilitates the fusion of the inner membrane, cristae formation, and unifying of compartments.

Mitochondria transduce energy from substrates through the tricarboxylic acid (TCA) cycle and the electron transport system (ETS) to generate ATP. The ETS consists of multipolypeptide complexes (I–V) embedded in the inner mitochondrial membrane (IMM) that receive electrons from reducing equivalents NADH and FADH_2_, generated by dehydrogenase activity in the TCA cycle. The electrons are transferred along the complexes with O_2_ serving as the final acceptor at complex IV [11] (Figure 2). The reduction potential (propensity to accept an electron) increases along the chain of complexes, and the energy generated is sufficient to drive the translocation of hydrogen ions across the IMM. This creates a proton gradient and membrane potential (collectively termed the proton motive force) that drives the synthesis of ATP as protons flow back to the matrix via complex V (ATP synthase). This process is also called oxidative phosphorylation (OXPHOS). However, the ETS is not a perfectly efficient system, and significant (and highly variable) proton “leak” occurs by the movement of hydrogen ions back into the matrix space that is not mediated through complex V. In this manner, proton transfer can be uncoupled from ATP phosphorylation, and this inefficiency contributes to the demand for reducing equivalents. Mitochondria are therefore thought to have a much greater capacity to generate ATP than what is usually required [12]. 

Because of this metabolic latitude, many assume that mild impairment in mitochondrial function *per se* does not cause cellular disturbances associated with aging and with chronic diseases such as insulin resistance and type 2 diabetes [12]. Indeed, whether mitochondrial dysfunction is a cause or a consequence of cellular impairment and the aging process is a subject of intense debate. Moreover, the definition of mitochondrial dysfunction itself has been the subject of controversy. For example, alterations in mitochondrial mRNA transcripts may not result in changes in protein levels, so it is not wholly clear whether this state—whether compensatory or not—can be counted as a disruption in normal function [13].

In this paper, we focus on the aging of skeletal muscle (SM) mitochondria, with an eye towards the putative role of mitochondria in SM aging. We look at several different aspects of mitochondrial function—which we define to mean any process that involves the mitochondria—and explore the evidence for dysfunction, which we define to mean any deviation from normal function.  Section 2 examines the impact of aging on the biochemical and bioenergetic pathways in SM mitochondria.  Section 3 explores the changes in mitochondrial and cellular dynamics, which may further exacerbate the age-related decline in muscular function. The review concludes in Section 4 with an overview of strategies to attenuate the aging of SM mitochondria.

## 2. Biochemical and Bioenergetic Aging of Mitochondria

During the aging process, mitochondria are characterized by changes in oxidative stress, a decay in mitochondrial DNA, a reduction in some enzyme activities, and alterations in mitochondrial respiration. These biochemical changes are accompanied by phenotypic changes in the mitochondria themselves. In old age, a significant proportion of the mitochondrial organelles are abnormally enlarged and more rounded in shape (reviewed in [14, 15]). An increasing proportion of them are also depolarized or nonfunctional, perhaps indicative of defects in mitochondrial turnover [15–17]. Within the mitochondria themselves, shortened mitochondrial cristae and vacuolization of the matrix are apparent, which lead to homogenization of the materials within the mitochondrial compartments [14, 18]. Coupled with these morphological changes, the density of mitochondria in SM substantially drops [13, 19–21], as shown for example, by electron microscopy in the *vastus lateralis* muscle of people over 60 years of age [20]. 

### 2.1. Increased Oxidative Damage: ROS Production and Scavenging

Within the cell, the mitochondria constitute the major source of reactive oxygen species (ROS). Mitochondrial complexes I and III are the main sites of superoxide generation and contribute the most to ROS production [22]. The reactive oxygen species, including O_2_^∙−^ and H_2_O_2_, can cause oxidative damage to surrounding structures and the particularly vulnerable mtDNA, which is in close proximity to the primary site of ROS production. Oxidation by ROS results in the synthesis of faulty proteins, oxidized lipids, and mtDNA mutations, which may lead to cellular and mitochondrial dysfunction. These processes are implicated in the mitochondrial theory of aging [23, 24], which holds that the accumulation of ROS damage over time leads to age-associated mitochondrial impairment. In general, ROS production is found to be increased in aged muscle in both the subsarcolemmal and intermyofibrillar pools of mitochondria [25] (though some report a decrease in ROS production with age [26]). This increase in ROS production is associated with oxidation of ETS complex V, leading to decreased ATP production [27, 28], increased levels of 8-oxodeoxyguanosine (8-oxoG) from DNA oxidation [25, 29], increased levels of protein carbonyls, and increased nitration [30]. In particular, proteomic studies have found increased nitration of complex II and altered carbonylation of complex I, complex V, and isocitrate dehydrogenase (reviewed in [31]).

**Figure 3:**
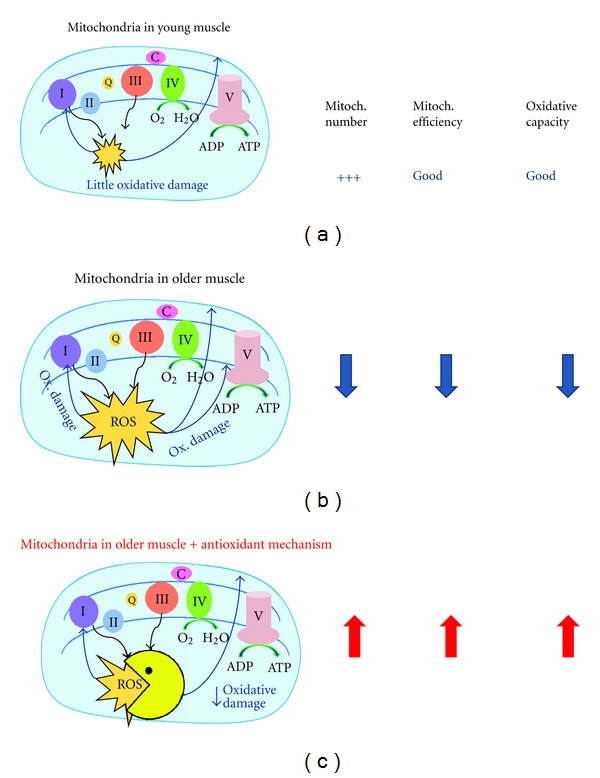
The electron transport system (ETS) of the inner mitochondrial membrane is the primary site of reactive oxygen species (ROS) production and therefore the main source of oxidative stress (damage to proteins, lipids, and DNA) in the mitochondria and in the cell. Free radical superoxide anions (O_2_^∙−^) are generated when electrons are donated from complexes I and III of the ETS to O_2_ instead of the appropriate ETS subunit. 2–4% of total oxygen consumption may go toward the production of ROS instead of energy as ATP. Scavenging enzymes represent an important mitochondrial defense mechanism against oxidative stress by neutralizing O_2_^∙−^ within the mitochondrial matrix (superoxide dismutase; MnSOD = SOD2) and catalyzing the reduction of mitochondrial SOD2-generated H_2_O_2_ to nontoxic H_2_O in the mitochondria and the cell (glutathione peroxidase and catalase). Mitochondria in young muscle (a) are numerous and efficient. With age (b), muscle mitochondria become less numerous and seem to develop impaired function associated with reduced oxidative capacity. Through lifestyle changes such as exercise, and caloric restriction, and caloric restriction mimetics, we hypothesize that antioxidative enzymes are upregulated, and that most of the above impairments in aged muscle may be improved (c).

Cumulative oxidative damage may in part be attributed to a reduction in ETS activity that would extend the length of time that electrons remain at complexes I and III, increasing the potential for donation of electrons to oxygen [32]. In theory, it could also be attributed to reduced activity of antioxidant defenses, including manganese superoxide dismutase (MnSOD), catalase (CAT), and glutathione peroxidase (GPx). These enzymes work together to convert O_2_^∙−^ to H_2_O_2_, which is then further reduced to H_2_O. Data on antioxidant enzyme activity and aging is mixed, with some studies showing increased activity [33–35] while others show reduced enzyme activity [36–38]. We recently found in a group of elderly adults aged 70–84 years that the expression of total superoxide dismutase (SOD) was unchanged compared to that of a group of young (20–34 years old) BMI-matched individuals; however, urinary isoprostanes (a marker of whole-body oxidative damage) was increased by 38% [10]. Furthermore, TCA cycle activity measured as citrate synthase (CS) activity was 19% lower, and mitochondrial capacity was 17% lower in the elderly, although mitochondrial content (OXPHOS protein) was unchanged. Safdar et al. recently showed that the protein content of MnSOD (mitochondria-specific) was significantly reduced in active and inactive older adults compared to their younger counterparts; however, MnSOD activity in the older subjects who were recreationally active was similar to that of young subjects [39]. Together, these data suggest that oxidative stress is increased with age without an accompanying increase in antioxidant activity and may be linked with mitochondrial dysfunction. Exercise and caloric restriction may alleviate the age-associated oxidative stress by upregulating antioxidant enzyme activity even in the face of reduced antioxidant protein content (Figure 3).

If oxidative stress contributes to mitochondrial dysfunction, can mitochondrial dysfunction be reversed by changing the cellular oxidative status? A recent study attempted to answer this question using a transgenic mouse model to express human catalase targeted to the mitochondria (MCAT) [40]. Mitochondrial function was assessed both *in vitro* and *in vivo* in wildtype young (3–6 months old) and older (15–18 months old) lean healthy mice versus age-matched MCAT transgenic littermates. Whereas the older wildtype mice exhibited all the “usual” deleterious metabolic impairments including increased oxidative damage, reduced mitochondrial content (~30%) and function, increased intramuscular lipid (~70%), and marked muscle insulin resistance (~35%), the older MCAT mice resembled the young mice and demonstrated none of the age-associated impairments. These data suggest that by increasing ROS scavenging and reducing the oxidized state of the cell, many age-related deficits can be prevented.

### 2.2. Increased Uncoupling?

Another mechanism to reduce oxidative stress may be mediated by attenuation of the proton motive force, thereby decreasing the potential for electrons to form oxygen radicals. This could be accomplished by increasing respiration or by increasing proton leak. Rodent studies on age-associated changes in proton leak are mixed. Iossa et al. [41] showed that proton leak was decreased with age, resulting in increased mitochondrial efficiency. Conversely, reduced coupling was found in aged mice using an *in vivo* approach to measure the P/O ratio [42]. Data in aged humans are scarce. We recently found that the *in vivo* P/O ratio determined by ATP turnover (demand) and oxygen uptake of the *vastus lateralis* was 21% lower in elderly compared to young adults [10], suggesting that less ATP was produced per oxygen uptake in the elderly adults. This lends support to earlier work by Amara et al. [43] showing that mild uncoupling protects against age-related declines in mitochondrial function and cellular ATP concentration. Furthermore, we found that a lower P/O ratio was associated with lower SOD activity, indicating a possible link between cellular antioxidant activity and mitochondrial coupling. In particular, increased oxidative stress with nonupregulated antioxidant defense may induce mitochondrial uncoupling in order to reduce the oxidative potential. This potential adaptive mechanism should be further explored.

### 2.3. Decay of Mitochondrial DNA

Age appears to affect both mitochondrial DNA (mtDNA) content and integrity. Mammalian cells typically contain on the order of 1,000–10,000 copies of mtDNA, which code for less than 10% of all mitochondrial proteins; the rest is encoded by nuclear DNA [44, 45]. At 16.6 kbp long, each double-stranded circular mtDNA molecule encodes 13 genes involved in the ETS (subunits of complexes I, III, IV, and V), 22 tRNAs, and 2 rRNAs [46]. A majority of studies [47–50], though not all [51], report that mtDNA copy number decreases with age in human SM and may be caused at least in part by oxidative damage, as the age-associated decline in mtDNA copy number tends to be greater in more oxidative fibers [52].

The decline in mtDNA is also accompanied by an increase in mtDNA damage, particularly deletions and oxidative lesions, but also point mutations, tandem duplications, and rearrangements (reviewed in [53, 54]; in particular [36–38, 48, 55–64]). mtDNA is particularly susceptible to oxidative damage because it is near the major site of ROS production, lacks protective histones, and has weaker DNA repair mechanisms [65, 66]. One study found that deletions affect up to 70% of mtDNA molecules in the SM of individuals aged 80 years and older [56]. However, there are disagreements over mtDNA mutation frequencies (reviewed in [54]) and to what extent ROS production is responsible; there is increasing evidence that perhaps a majority of mutations are due to the inherent error rate of mtDNA polymerase gamma (Pol gamma) [67].

The functional impact of mtDNA damage is still a matter of debate both in general (reviewed in [54, 67, 68]) and in the context of sarcopenia (reviewed in [69]). Is mtDNA damage a consequence or a cause of the aging process? On one hand, the onset of a mitochondrial decline in energy production occurs before mtDNA mutations are often detectable [70]. On the other hand, several studies in humans reveal strong correlations between mtDNA mutation rates and bioenergetic deficiency (typically complex IV) or muscle fiber atrophy [43, 55, 62, 63, 71–74]. In addition, specific point mutations cluster at mtDNA replication control sites, which may reduce gene transcription and be at least partially responsible for declining levels of protein synthesis with age [60, 75]. Other evidence comes from mtDNA mutator mice, which are genetically engineered to have defects in the proofreading function of Pol gamma. They accumulate mtDNA mutations at an accelerated rate, have more abnormal mitochondria, and exhibit premature aging, sarcopenia, and reduced lifespan [76–78].

### 2.4. Alterations in Mitochondrial mRNA and Protein Levels

The expression of most mitochondrial genes, including cytochrome c [79], does not change with age. However, declines in gene transcripts are observed for several of the polypeptide components of complexes I, IV, and V, whereas for complexes II and III, declines are evident for only a couple components with most being unchanged [73, 80–84]. In addition, the transcripts of some nuclear genes encoding mitochondrial regulatory proteins, a few TCA proteins (including one transcript variant of citrate synthase), and some glycolysis enzymes appear to be altered in aged human SM [48, 52, 80, 83, 84]. Many similar changes in gene expression (or lack thereof) have been observed in monkeys and rats [52, 85–87]. However, there are discrepancies among reports, which are at least in part explained by muscle-specific differences in gene expression [52, 88]. Whether these alterations in gene transcripts of energy-producing pathways affect muscle aging is unclear. While much evidence points to a functional role, Giresi et al. found that the key genes associated with sarcopenia are involved in inflammation, apoptosis, and regulating mRNA splicing, not in OXPHOS [89].

Of course, age-related alterations in mRNA levels may not induce similar changes in protein abundances, which are controlled by the balance between synthesis and degradation [13, 90]. Both mitochondrial protein synthesis [75] and proteolysis via ubiquitin-proteasome and lysosomal degradation systems (reviewed in [91, 92]) are known to decline with age. The majority of mitochondrial proteins, including polypeptide components of the ETS complexes, have not been found to change with age [10, 93]. Notable exceptions include complex II, which tends to increase with age at least in rodents [86, 94, 95], and complex IV, which tends to decrease with age in both humans [50, 96, 97] and rodents [86, 98]. This is supported by observations of increased complex IV-deficient and complex II-hyperactive fibers with age [99–101]. For complexes I, III, and V, proteomic analysis shows that the occurrence of abnormal polypeptides tends to increase with age in both human [102] and rodent SM [86, 94, 98, 103], but is decreased in human studies using other approaches [48, 50, 96]. Proteins involved in glycolysis tend to be unchanged or reduced with age, which is consistent with the observed shift from glycolytic to more oxidative metabolism, while the data on TCA proteins (in particular, citrate synthase and isocitrate dehydrogenase) is mixed [48, 50, 86, 94–96, 98, 102, 103].

### 2.5. A Decline in Mitochondrial Energy Production?

It is unclear whether these age-related changes in turn affect mitochondrial energy production. When physical activity is not a criterion in subject selection, substantial declines in enzyme activity are often found to occur with age. Most such studies in human SM report that complex I and complex IV activities decrease substantially, perhaps because these two complexes have more of their subunits encoded by the more vuinerable mtDNA than the other complexes [1, 21, 56, 58, 75, 79, 90, 97, 104, 105]. Similar results have been reported in rodents and dogs [2, 5, 52, 106–112], though there have been exceptions both in humans [93, 113, 114] and rodents [86, 94, 109]. The activity of complex II, which is encoded entirely by the nuclear genome, appears not to change with age in either human [1, 56, 64, 113, 114] or animal [2, 86, 108] SM, with a few exceptions [39, 79, 94, 105]. Data regarding complex III activity is both less available and more mixed, with some studies reporting no change in human [113, 114] or animal [2, 108] SM, but others reporting a decrease in activity [5, 56]. Interestingly, one study found evidence of an age-related decline in complex III activity in females but not males [90]. Finally, the activities of enzymes involved in the TCA cycle and glycolytic pathways tend to be unchanged or decline with age, and in some cases (e.g., citrate synthase), the trend is not clear [10, 21, 26, 39, 48, 56, 75, 93, 96, 97, 104, 105, 111–113, 115–120]. Some of these discrepancies may be explained by the differential impact of aging on different muscles. For example, one study in rats found a decrease in complex IV activity in the *lateral* but not *medial gastrocnemius* [52], and a study in humans found that citrate synthase activity was negatively correlated with age in the *gastrocnemius* but not the *vastus lateralis* [121]. Other inconsistencies may be explained by differences in isolation or techniques, or in the normalization of enzymatic activity. For example, Picard et al. found that measurements on isolated mitochondria tend to exaggerate the declines in mitochondrial function in comparison to measurements in intact mitochondria in permeabilized myofibers [86].

Enzymatic changes may in turn affect mitochondrial respiration and ATP flux. Short et al. found that the maximal capacity for ATP synthesis drops by 8% per decade or 5% when normalizing to mitochondrial protein content [48]. Similar results concerning mitochondrial respiration were reported by Trounce et al. [122]. But other human studies have found no evidence of a decline in mitochondrial respiration [26, 93, 115, 117], and there is no consistent pattern regarding how the respiratory control ratio—the ratio of states III and IV respiration activities—changes with age. Similarly, there are conflicting reports on fatty acid-linked mitochondrial respiration including reduced carnitine palmitoyltransferase-dependent and -independent pathways with age (particularly nonphosphorylating respiration [48, 86, 108]), while others see no age-related changes [26, 115, 117]. *In vivo* techniques have also been used to measure oxidative capacity. *In vivo* measurements of SM oxidative activity are typically performed using phosphorous magnetic resonance spectroscopy (^31^P-MRS) to probe the kinetics of phosphocreatine and its recovery time following muscle contraction. Some *in vivo* studies in older humans [10, 20, 118, 120], but not all [119, 123, 124], and at least one in mice [125] have found evidence of reductions in maximal ATP flux. In particular, Conley et al. found that elderly adults have a 50% reduction in oxidative capacity per muscle volume and a 30% reduction per mitochondrial volume [20]. An MRS study on basal ATP flux in humans, however, found no decline in flux with aging [43].

However, it has become increasingly clear that most of the declines in mitochondrial function attributed to chronological age are instead a result of physical inactivity. When physical activity levels are matched between young and old subjects or physical activity is otherwise taken into account, most studies find no age-related changes in mitochondrial enzyme activities, mitochondrial respiration, or ATP flux [13, 39, 50, 101, 126–129]. Interestingly, those studies that do report age-related declines even after matching for activity levels tend to involve *sedentary* young and old subjects [104, 114, 130], indicating that declines in function with aging may occur predominantly in sedentary individuals. In particular, in *ex vivo* studies, the activities of ETS enzymes and citrate synthase and mitochondrial respiration show strong dependences on physical activity level that are independent of age [101, 131, 132]. Similar results are also reported in mice [98, 133]. *In vivo* MRS studies on activity-matched subjects also tend to find no evidence of a change in mitochondrial oxidative capacity—in this case, maximal ATP flux—between young and old subjects [134–136]. However, one *in vivo* MRS study in activity-matched sedentary individuals did report a reduction in basal oxidation and phosphorylation [130]. Also, glycolytic flux is lower in older activity-matched people [135, 136], and a recent MRS study showed that oxidative capacity does indeed change in some muscles with age but also demonstrated that physical activity is intimately linked with oxidative capacity [128]. Interestingly, there is evidence that physical activity can reverse the age-related declines in most but not all mitochondrial markers of energy production [50, 132], which while encouraging, nonetheless indicates that there are residual declines in a small subset of markers that cannot be completely prevented.

## 3. Age-Related Changes in Mitochondrial Dynamics

### 3.1. Decreased Mitochondrial Biogenesis

Once thought of as relatively static round organelles, mitochondria are now recognized as highly dynamic, existing in networks that are constantly being remodeled by biogenesis, fusion and fission, and degradative processes such as autophagy. Through these dynamics, they both respond to and drive cellular processes, including apoptosis, whose dysregulation is thought to be a key factor in sarcopenia.

Mitochondrial biogenesis is the expansion of existing mitochondrial content—whether through growth of the mitochondrial network (increase in mitochondrial mass) or division of preexisting mitochondria (increase in mitochondrial number; also discussed later in this paper). It is triggered when the energy demand exceeds respiratory capacity—in particular, in response to exercise, stress, hypoxia, nutrient availability, hormones (including insulin), ROS production, and temperature (reviewed in [137, 138]). Once biogenesis is triggered, the nuclear genome produces mitochondrial regulatory factors, which are then imported into the mitochondria to initiate replication and transcription of mtDNA, ultimately resulting in expansion of the mitochondrial network. The initial triggers are thought to arise from signaling cascades involving the energy sensor AMP kinase (AMPK) and/or alterations in Ca^2+^ flux and protein kinases such as calcium/calmodulin-dependent kinases (CAMKs), protein kinase C (PKC), and p38 MAPK (particularly in response to muscle contraction) (reviewed in [138]). These signals in turn induce the expression of the peroxisome proliferator-activated receptor (PPAR) gamma coactivator (PGC-1) family of cotranscription factors, particularly PGC-1*α*, which is also directly activated by AMPK [139] and p38 MAPK [140–142], as well as many key signaling molecules like SIRT1 [143, 144] and transducers of regulated CREB (cAMP response element-binding protein)-binding proteins (TORCs) [145].

As the master regulator of biogenesis, PGC-1*α* coordinates and cooperates with multiple cotranscription factors—including PPARs, myocyte enhancing factors (MEFs), and CREB—to induce the transcription of nuclear genes encoding mitochondrial proteins [146, 147]. Most importantly, PGC-1*α* activates the nuclear respiratory factors 1 and 2 (Nrf-1, Nrf-2) on the promoters [148, 149], thereby driving the transcription of an even greater assortment of nuclear-encoded mitochondrial proteins (reviewed in [137, 138, 150]), including mitochondrial transcription factor A (Tfam). Tfam controls mtDNA transcription and content and organizes mtDNA into nucleoid-like structures, which are thought to maintain mtDNA integrity [151]. The Tfam precursor protein, along with numerous other mitochondrial precursor proteins, are targeted to the mitochondria by chaperones and then imported via the mitochondrial protein import machinery (reviewed in [152–155]). Import is accomplished through a set of aqueous pores formed from translocases of the outer membrane (TOMs; e.g., Tom20) and translocases of the inner membrane (TIMs) through pathways that depend on whether the polypeptide is destined for the outer mitochondrial membrane (OMM), IMM, or mitochondrial matrix. Once imported, the precursor proteins are modified, folded, and assembled into their final form. In particular, Tfam can then act on mtDNA driving its replication through Pol gamma and its transcription through mitochondrial RNA polymerase [156]. Finally, the translated ETS polypeptides from the nuclear and mitochondrial genomes are assembled into multisubunit enzymes.

With increasing age, the density of mitochondria in SM drops substantially [13, 19–21, 25], suggesting an overall decline in mitochondrial biogenesis. Moreover, there is an impairment in AMPK-stimulated biogenesis in old age [157]; however, the reasons why are largely unknown. Declining PGC-1*α* levels could explain the reduction in mitochondrial biogenesis, as overexpression of PGC-1*α* in the SM of aged mice improved oxidative capacity, suppressed mitochondrial degradation, and prevented muscle atrophy [158]. These improvements were accompanied by attenuation of the age-related increase in inflammatory cytokines and by prevention of insulin resistance [158]. However, measurements of PGC-1*α* in aged SM are not definitive: one study found a decline in protein abundance in rats [25], and some studies of gene expression have reported reduced expression [96, 112, 157], while others found no change [10, 159]. Similarly, reports on the gene and protein expressions of both Nrf-1 and Tfam are conflicting [25, 50, 80, 96, 159, 160]; however, preliminary evidence suggests that Nrf-1 binding to the Tfam promoter appears to increase in old age [160].

### 3.2. Changes in Fission and Fusion?

Mitochondrial dynamics are also influenced by the balance between fission and fusion (Figure 2(b)). Fission, or division, of mitochondria is required to transmit mitochondria among dividing cells and to meet increased ATP demands. Fission also plays a key role in maintaining mitochondrial quality and mtDNA integrity, as it allows dysfunctional mitochondria to be severed from the network and to be removed by autophagy [161, 162]; indeed, mitochondria excised by fission often have a lower membrane potential, a target for autophagy [16]. In mammals, fission is known to be orchestrated by two proteins: Fis1 and the GTPase dynamin-related protein Drp1. Fis1, which localizes in the OMM, recruits the cytosolic protein Drp1 [162, 163]. Once recruited to the OMM, Drp1 wraps around and constricts the membrane, initiating fission (reviewed in [164]). One study found that activation of the fission machinery was sufficient to induce muscular atrophy [165], while another study found that in yeast, increased fission leads to a shortened lifespan and a greater sensitivity to ROS-induced apoptosis [166]. Because Fis1 protein levels are elevated in aging rat SM [94] and Drp1 transcripts tend to be lower in older humans [21], age-associated increases in fission may indeed contribute to sarcopenia. On the other hand, suppression of Fis1 or Drp1 produces elongated mitochondrial networks and a senescent phenotype but increases ROS production and mtDNA damage [163, 167, 168].

The counterpart of fission is fusion, which unifies mitochondria. Fusion allows mixing of mitochondrial compartments, facilitating equilibration of OXPHOS proteins, energy exchange, and complementation of the mitochondrial genome [169, 170]. In aging, this mixing is thought to prevent mutations in mtDNA from resulting in respiratory dysfunction; however, it also permits the accumulation of mutated mtDNA that might otherwise be removed via fission and autophagy [170, 171]. For these reasons, fusion plays a role in regulating mtDNA integrity and respiratory function [172–174]. In humans, fusion is known to be controlled by optic atrophy 1 (Opa1) and the two GTPases mitofusin 1 and 2 (the isoforms Mfn1 and Mfn2). Mfn1 and Mfn2 are located in the OMM, where they promote tethering and fusion [162], while Opa1 facilitates fusion from its localization on the IMM (reviewed in [164]). Opa1 also assists in regulating degradative processes: it regulates apoptosis by keeping the inner mitochondrial cristae junctions tight to prevent cytochrome c release, which triggers apoptosis [175, 176], and its cleavage may be involved in flagging dysfunctional mitochondria for autophagic removal [176]. One study reported that Mfn2 gene expression was lower in the SM of older humans [21]. Interestingly, muscle-specific Mfn1- and Mfn2-knockout mice experience enhanced mitochondrial proliferation and increased mutations in and depletion of mtDNA; these changes occur in parallel with accelerated muscle loss [177]. A mutation in the other key fusion protein, Opa1, is associated with reduced oxidative phosphorylation and ATP production in human SM [178]. Thus, age-associated changes in the dynamical remodeling processes of fission and fusion likely affect mtDNA integrity, respiratory function, ROS production, and cellular senescence.

### 3.3. Alterations in Mitochondrial Turnover

Mitochondrial turnover is executed predominantly by the autophagy-lysosome system, a cellular housekeeping system that degrades mitochondria as well as other cellular components. Removal of mitochondria through the autophagy-lysosome system is known as “mitophagy.” In mitophagy, dysfunctional mitochondria are recognized and engulfed in a double-membrane structure called a phagophore or preautophagosome. Once the engulfing process is complete, vesicles called autophagosomes form. The autophagosomes then fuse with the lysosome, producing autolysosomes, and the contents are hydrolytically degraded and recycled (reviewed in [16, 179, 180]). The process is mediated through a collection of several autophagy gene (Atg) products. In yeast, the recognition process is enacted through Atg32, a mitophagy-specific receptor on the OMM [181], and through other key players, such as Atg8 and its activator Atg7. Mitophagy in mammals has been less well characterized, but the mammalian homologues to Atg32 and Atg8 are believed to be Nix and LC3, respectively [180, 182]. In addition, mitophagy in mammalian cells may be carried out through ubiquitination of OMM proteins, followed by recognition via an LC3 complex (reviewed in [180]).

Relative to other mitochondrial dynamics, there is less known about the role of mitophagy in the aging of SM. Evidence suggests that mitophagy selectively removes defective mitochondria that are depolarized or produce excessive ROS (reviewed in [16, 17, 179, 183]). In corroboration, suppression of autophagy results in increased ROS production, reduced oxygen consumption, and higher mtDNA mutation rates [184, 185]. With age, autophagy has been found to decline both in general (reviewed in [186]) and in the SM of aged rats [187]. While the repercussions are still unknown, mitophagy has been negatively correlated with oxidative damage and apoptosis [187], suggesting that reduced autophagy rates may contribute to muscular dysfunction. This is confirmed by studies on muscle-specific Atg7 knockout mice, who accumulate abnormal mitochondria, have lower resting oxygen consumption, and experience increased oxidative stress and higher rates of apoptosis; these mice also suffer from muscle atrophy, weakness, and myofibril degeneration [188, 189]. Furthermore, studies in a few species indicate that enhanced autophagy may increase lifespan (reviewed in [190]).

Mitochondrial quality control and degradation are also modulated by the ubiquitin-proteasome system, which removes oxidized proteins and short-lived proteins (reviewed in [191]). Evidence from studies in mammals has suggested that ubiquitin-proteasome activity declines with age in SM and may contribute to muscular atrophy (reviewed in [91, 192]). However, aging may differentially affect the components of the ubiquitin-proteasome system [193], as some genes involved—including some proteasome subunits and ubiquitin-specific proteases—are expressed at higher levels in SM from older humans, yet others are unchanged or decreased with age [49, 84, 94, 194–196]. Finally, changes in proteasomal activity may be fiber type-specific [197]: in particular, one study in humans reported that ubiquitin protein levels increase in fast-twitch muscle fibers, which may explain why type II fibers atrophy faster with age [198].

### 3.4. Increased Mitochondria-Mediated Apoptosis

Mitochondria also respond to and modulate cellular dynamics through apoptotic signaling, which is activated when either OXPHOS or their redox potential is disrupted, or in response to proapoptotic signals (reviewed in [199]). Mitochondria can induce apoptosis through either caspase-dependent or caspase-independent signaling mechanisms (reviewed in [199–201]). Caspase-dependent signaling is dependent on the release of cytochrome c from within the mitochondria, which triggers a cascade of actions by cysteine proteases called caspases that results in apoptosis. In brief, cytochrome c and other proapoptotic factors are released from the mitochondria. Once released, cytochrome c joins with apoptotic protease activating factor-1 (Apaf-1) and procaspase-9 to form a complex called the apoptosome. The apoptosome then cleaves and activates procaspase-9, which acts on caspase-3. Caspase-3 in turn activates a caspase-activated DNase (CAD) to degrade DNA and initiates cellular degradation. In the caspase-independent pathway, which is also known as “mitoptosis,” the mitochondria release apoptosis-inducing factor (AIF) and endonuclease G (EndoG), which then induce chromatin condensation and DNA fragmentation. There are multiple points in these pathways that are regulated by pro- and anti-apoptotic proteins. In particular, the release of apoptotic triggers appears to be modulated through two mechanisms: (1) the balance of proapoptotic (e.g., Bax) and anti-apoptotic proteins (e.g., Bcl-2), particularly from the Bcl-2 family, which control OMM stability and form the mitochondrial apoptosis-induced channel (MAC), and (2) the mitochondrial permeability transition pore (mPTP). One example of the direct connection between mitochondrial and cellular dynamics is that mitochondrial fragmentation occurs around the time that proapoptotic factors are released from the mitochondria, and this step is contingent upon increased fission through Drp1 as well as a block in mitochondrial fusion (reviewed in [202]).

Apoptosis increases significantly with age and likely contributes to sarcopenia and other age-associated declines (reviewed in [201, 203, 204]). Though it is difficult to prove this conclusively, many studies have correlated rates of apoptosis with markers of sarcopenia or SM function (reviewed in [201, 203, 204]). In humans, the percent of apoptotic cells tends to increase with age, though generally more so in type II fibers [194, 205, 206]. The weight of evidence from both human and animal studies suggests that the caspase-independent pathway is upregulated with age, while the caspase-dependent pathway is not (reviewed in [201]). In particular, one study found an increase in AIF gene transcripts in SM from older people, but no change in Bax, Bcl-2, or caspase-3 expression [207], and another study in humans reported no change in caspase-3 or -7 activity [194]. This is also supported by animal studies, which have found that the mPTP becomes more susceptible towards being opened [25, 208, 209] and that the levels and activities of caspase-independent apoptotic proteins AIF and EndoG increase [25, 112, 210–213] with age. Recent evidence in rats suggests that apoptotic susceptibilities and markers are age- and fiber type-specific, which may explain some of the mixed results, particularly in regard to the caspase-dependent pathway [25, 214–216]. Interestingly, a study showed that disuse atrophy increased caspase-3 activity in young rats but not old and dramatically increased EndoG levels in old rats but not young, indicating that older SM likely responds to apoptotic stimuli through different signaling pathways than younger SM [212].

## 4. Strategies to Attenuate Mitochondrial Aging 

### 4.1. Exercise

Exercise training has long been known to induce mitochondrial biogenesis, upregulate SM gene expression and protein synthesis, and increase SM oxidative capacity [217, 218]. It is apparent from previous research that physical activity decreases during aging [219]. Therefore, it remains unclear whether the abnormalities in mitochondrial function are a primary effect of aging or are due to the associated decline in physical activity. This issue is compounded by the cross-sectional nature of some studies without objective control for activity level [130, 220–222]. For example, we recently found that mitochondrial capacity was reduced in elderly subjects compared to their young BMI-matched counterparts [10]. In this study, we included only sedentary individuals, defined as less than 2 hours of intentional physical activity per week. Despite careful screening for activity levels using questionnaires, we found that daily physical activity was significantly lower than reported in the elderly group. This suggests that independent of exercise training, simply living an active lifestyle may have a significant impact on mitochondrial function; however, we cannot determine the cause and effect due to the cross-sectional nature of the analysis.

There is strong evidence that exercise training can improve SM mitochondrial function in elderly adults [84, 96, 97, 223–225] and may also protect against age-associated apoptosis [226]. Short et al. found that 4 months of aerobic exercise in older adults increased protein synthesis, mitochondrial enzyme activity (citrate synthase and cytochrome c oxidase), and expression of genes involved in mitochondrial content and biogenesis to levels similar to those in younger adults [97, 227]. In a separate study, 12 weeks of aerobic exercise training increased mitochondrial content and activity, particularly in the subsarcolemmal fraction [224]. Exercise has also been shown to increase the activity of antioxidant enzymes and heat shock proteins [228], potentially reducing ROS production and decreasing the potential for mitochondrial oxidative damage thought to occur during aging. Indeed, Parise et al. showed that resistance exercise in older adults increased antioxidant content (but not activity), decreased oxidative damage (8-OHdG), and increased complex IV activity [229, 230].

Despite these significant differences in mitochondrial parameters, most data show that some impairment remains. For example, the usual age-associated decline in mitochondrial oxidative capacity was absent in older adults who were chronically endurance trained. However, chronic exercise did not completely restore the expression of mitochondrial proteins, mtDNA content, and mitochondrial transcription factors to the levels of younger subjects, suggesting a persisting, independent effect of age [50]. Supporting this, Melov et al. used an “omics” approach to show that after 6 months of exercise training, the transcriptional signature of aging was substantially but incompletely reversed back to the transcriptome of younger adults [84]. In all, the results to date indicate that exercise can help to attenuate age-associated changes in SM mitochondria. However, it does not completely prevent these changes. The data available are rather limited and apply mostly to short-term exercise interventions (i.e., 12–24 weeks) rather than chronic exercise. In addition, the impact of active lifestyle changes—for example, decreasing sedentary time and increasing “nonexercise” activity—on mitochondrial function in elderly adults may be significant [231] and needs further investigation, as this may be a more logical approach rather than prescribing an exercise regimen.

### 4.2. Caloric Restriction

Caloric restriction, which typically involves consuming 20–40% fewer calories than normal, also preserves mitochondrial health and attenuates SM decline with age. Caloric restriction (CR) is recognized as the most robust intervention that retards both primary aging (natural age-related deterioration) and secondary aging (accelerated aging due to disease and negative lifestyle behaviors), thereby increasing both median and maximal lifespan in many species. While CR studies in primates and humans are largely ongoing, studies in rodents consistently show that CR extends maximum life span by up to 50% and reduces the incidence of many age-associated diseases, including cancer and metabolic diseases (reviewed in [232]). Preliminary evidence in rhesus monkeys indicates similar effects can be expected in primates [231].

The benefits ascribed to CR are believed to be due in large part to reductions in oxidative stress (reviewed in [233]). In primates, a decade-long CR intervention resulted in marked decreases in oxidative damage to lipids and proteins [234]; in aged rats, CR also reduces ROS production [159, 235, 236]. As a result, aged CR animals exhibit fewer mtDNA and nuclear DNA mutations and less oxidative damage to SM mitochondria than their ad libitum-fed counterparts [99, 236–240]. Some of these findings have been replicated in the still ongoing CR trial in humans, dubbed CALERIE, which reported that CR subjects had less mtDNA damage and more mtDNA content than controls [241]. Microarray and RT-PCR experiments confirm that CR increases transcripts of genes involved in ROS scavenging, including SOD and GPx, and decreases transcripts from stress response genes [196, 242].

Calorie restriction appears to modulate mitochondrial efficiency, content, and function. CR lowers energy expenditure in animals and humans by producing mitochondria that consume less oxygen yet are able to maintain normal levels of ATP production (reviewed in [232]; in particular, [159, 237, 241–243]). It is generally believed that this energetic adaptation is mediated via decreased proton leak, which has been confirmed in rodent studies, and that decreased proton leak is in turn enabled by the shift to a less oxidative milieu [159, 237, 244]. In terms of mitochondrial content and function, CR does not affect the gene expression, protein level, or activity of citrate synthase, nor the activities of other TCA proteins [111, 112, 241, 242, 245]. However, CR may affect some ETS enzymes. CR reduced the age-associated accumulation of complex IV-negative and complex II-hyperactive fibers in rats [99] and in rhesus monkeys [100]. Only complex IV activity, however, is consistently responsive to CR, showing increased activity in comparison to that in aged rodents and usually similar activity to their younger counterparts [110–112, 246]. Nonetheless, CR may prevent the decline in the activities of complexes I–III in some muscles [111]. However, a study in rhesus monkeys found that about 9 years of CR only attenuated the decline in gene expression of the complex II iron-protein subunit [85], and a 14-week CR intervention where the rats were fed at 70% of ad libitum levels found no increases in any mitochondrial gene transcripts or proteins [245]. CR rodents did have fewer ETS abnormalities, but the abnormalities were otherwise similar to controls, suggesting that CR only affects the onset of fiber atrophy [240, 247]. However, in primate studies, CR did not alter the number of fibers with ETS abnormalities, but nonetheless preserved muscle fiber [100, 248].

CR also affects mitochondrial dynamics. In rodent SM, CR also increases mitochondrial biogenesis relative to controls by slowing the decline in PGC-1*α* gene expression with age, which may be at least partially responsible for maintaining oxidative capacity in aged CR animals [110, 112]. The CALERIE study in humans also reported increased transcripts from several genes involved in mitochondrial biogenesis, including PGC-1*α*, Tfam, and SIRT1 [241]. It is not clear, however, whether CR affects the mitochondrial metabolic regulator AMPK, as there have been mixed results [249, 250]. In addition, CR attenuated the decline in mitophagy in the SM of old rodents [187] and the decline in ubiquitin-proteasome activity in monkeys [251]. It also reduced apoptosis susceptibility by promoting a remodeling of caspases and caspase-related proteins to favor decreased likelihood of cytochrome c release from the mitochondria [187, 252–254]. These findings are particularly important as mitochondrial dysfunction and apoptosis have been proposed as key mediators of sarcopenia.

Combined, these results suggest that CR reduces oxidative stress and remodels mitochondrial dynamics to promote the production of more fuel-efficient mitochondria that produce less ROS and to favor the removal of dysfunctional mitochondria [187, 243, 255]. Regardless of the underlying mechanism, CR has repeatedly been shown in rodents to either partially or fully oppose the hallmarks of sarcopenia, including the age-related declines in muscle force, fiber cross-sectional area, fiber number, and fiber type (reviewed in [99, 247, 254, 256–258]). Similar findings have been reported in CR studies in rhesus monkeys [100, 248].

### 4.3. Mimetics

Currently, there is strong interest in using a CR- or exercise-mimetic to attenuate age-related mitochondrial dysfunction. The best known of these is resveratrol (3,5,4,9-trihydroxystilbene), a phytoalexin that is abundant in red wine. In the context of secondary aging, resveratrol's effects on mitochondria and SM have been well studied. In rodent models involving metabolic pathology, resveratrol improves mtDNA copy number and function, increases mitochondrial biogenesis, improves exercise capacity and motor function, and mitigates metabolic dysfunction [259–261]. On a molecular level, resveratrol induces an increase in the expression of PGC-1*α*, Tfam, and UCP3, and increases SIRT1, AMPK, and PGC-1*α* activation [259, 260]. In humans, a recent 30-day study of resveratrol supplementation in 11 obese but metabolically healthy men reported improved mitochondrial respiration in the presence of fatty acid-derived substrate, increased AMPK and citrate synthase activity, and higher SIRT1 and PGC-1*α* protein levels; however, no changes in mitochondrial content were observed [262]. Interestingly, while resveratrol was originally identified as a potent activator of SIRT1, there is controversy surrounding its mode of action, and it may exert much of its effects through AMPK [260, 263, 264].

Rodent studies consistently show improved mitochondrial health with resveratrol (reviewed in [265]). For example, in senescence-accelerated prone mice, those given resveratrol had higher physical endurance, maximal force contraction, and oxygen consumption, and higher levels of transcripts from PGC-1*α* and ETS genes [266]. Aged rats given resveratrol exhibited similar responses; they also displayed reduced levels of oxidative stress but no changes in most apoptotic markers, indicating the improvements were mediated through changes in redox status and not apoptotic pathways [267]. Like CR, resveratrol's effects on mitochondrial biogenesis are believed to be mediated in large part via reductions in mitochondrial ROS production and via upregulation of fatty acid catabolism coupled with a downregulation in fatty acid synthesis [137]. However, one recent study of long-term resveratrol supplementation in mice did not find any improvement in the age-related declines in PGC-1*α*, mitochondrial content, muscle mass, and maximal isometric force production; though resveratrol did preserve type II fiber contractile function and reduce oxidative stress [268].

## 5. Conclusions

In summary, animal and human data consistently show that skeletal muscle mitochondria are altered in aging, including increased mutations in mitochondrial DNA, decreased expression of some mitochondrial proteins, reduced enzyme activity and altered respiration with reduced maximal capacity in sedentary adults, and reduced total mitochondrial content with increased morphological changes. Since the primary role of mitochondria is to produce ATP to maintain the energy status of the cell, shifts in respiratory activity and capacity can lower the membrane potential, reduce cellular ATP concentration, and signal cellular apoptotic events. Increased apoptosis without correspondingly increased protein synthesis will eventually lead to net muscle fiber loss. All of these factors probably contribute to age-associated sarcopenia, and mounting evidence suggests that most of these age-related changes can be either prevented or attenuated through increased physical activity. 

There is some thought that the accumulation of oxidative damage caused by long-term ROS production is responsible for these changes with age. Indeed, blocking ROS formation by the targeted expression of antioxidant enzymes ameliorates age-associated dysfunction and returns mitochondrial parameters to those of young animals (Figure 3). Whether this is also true for humans is unknown; however, strategies that improve mitochondrial function, such as exercise and caloric restriction, also reduce ROS production and increase antioxidant defenses. Exercise is also known to be a powerful stimulant of mitochondrial biogenesis and oxidative capacity, although exercise training in elderly adults probably does not completely reverse the primary effects of aging. However, exercise training—even adopting active lifestyle habits—may clearly reduce the rate of mitochondrial decline and attenuate the aging phenotype. Whether CR- and exercise-mimetics, including resveratrol, work as effectively remains to be determined and is an area of active research.

In conclusion, there is clearly a need for more research in this field and particularly to:

- figure out which age-related changes are universal and which depend on physical activity or lifestyle habits or gender;
- unravel how cells compensate for oxidative damage and mitochondrial dysfunction;
- elucidate how fusion, fission, and autophagy work together to ensure quality control and to remove defective mitochondria; and
- determine which mitochondrial declines can be slowed down (or even reversed) by healthier lifestyles.

We expect this to continue be a fruitful and exciting area of study in years to come.
